# A homozygous variant of *WDR45B* results in global developmental delay: Additional case and literature review

**DOI:** 10.1002/mgg3.2036

**Published:** 2022-08-13

**Authors:** Jinhong Zhang, Yan Lu, Xiaoyu Tian, Xinyi Men, Yange Zhang, Huifang Yan, Fan Yang, Zuozhen Yang, Xiuxia Wang

**Affiliations:** ^1^ Department of Pediatrics The Second Hospital of Hebei Medical University Shijiazhuang Hebei China; ^2^ Department of Pediatrics Hengshui people's Hospital Hengshui Hebei China; ^3^ Cipher Gene LLC Beijing China

**Keywords:** clinical heterogeneity, global developmental delay, Trio‐WES, *WDR45B*

## Abstract

**Background:**

Global developmental delay (GDD) has a heterogeneous clinical profile among patients, accounting for approximately 1%–3% of cases in children. An increasing number of gene defects have been demonstrated to be associated with GDD; up to now, only limited studies have reported developmental disorders driven by WDR45B.

**Methods:**

Trio‐whole exome sequencing (Trio‐WES) was performed for the patient and her family. All variants with a minor allele frequency <0.01 were selected for further interpretation according to the ACMG guidelines. Candidate pathogenic variants were validated by Sanger sequencing in her family.

**Results:**

A homozygous nonsynonymous variant in *WDR45B* [NM_019613.4: c.677G>C (p. Arg226Thr)] was identified from the proband. The variant was absent in published databases such as gnomAD and Exome Aggregation Consortium (ExAC). The variant was predicted to be damaging for proteins and classified as VUS according to the ACMG guidelines. We reviewed the literature, and the development delay level in our case was less severe than the other reported cases.

**Conclusion:**

We reported another case with a novel homozygous variant of *WDR45B* and showed the heterogeneity of clinical features.

## Graphical abstract

As a rare pathgenic gene, only 8 variants were reported for WDR45B. we report another new case with novel variant and clinical features.

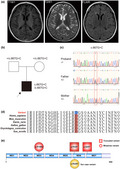

## 1 INTRODUCTION

Approximately 52.9 million children were reported to have developmental delays in 2016, and 95% of that population resides in low‐ and middle‐income countries (LMICs) (Khan & Leventhal, 2021). According to statistics from the World Health Organization (WHO), approximately 10% of the total population of children suffered disabilities (Dornelas Lde et al., 2015). Global developmental delay was a subgroup of developmental disorders which included more than two developmental disorders: gross/fine motor, speech/language, cognition, social/personal, and activities of daily living. Therefore, the clinical features of global developmental delay exhibited a heterogeneous etiologic profile (Shevell et al., 2003). Notably, Global developmental delay (GDD) also accounted for 1% to 3% of deaths in children (Khan & Leventhal, 2021).

WDR45B and WDR45 belong to the WIPI or SVP1 family of WD40 repeat‐containing proteins. They contain seven WD40 repeats that can fold into beta‐propeller structures and mediate protein–protein interactions, as well as a motif in which they interact with phospholipids. Variants in WDR45 and WDR45B had been reported to drive the human neurological diseases beta‐propeller protein‐associated neurodegeneration (BPAN) (Haack et al., 2012; Saitsu et al., 2013) and intellectual disability (ID) (Anazi et al., 2017), respectively. Knockout (KO) mice of *WDR45* and *WDR45B* demonstrated impaired cognitive function and pathological changes in the central nervous system (CNS) (Ji et al., 2020).

To date, variants in *WDR45B* are rare, and clinical features are relatively heterogeneous. Only two homozygous nonsense variants (c.799C>T (p. Q267*); c.673C>T (p. R225*)), three splicing variants (c.619‐3A>G; c.427+4A>G; c.67+1G>T), one frameshift variant (c.428‐10_435del18), and two missense variants (p. R109Q; p.R225Q) of *WDR45B* have been identified in 11 families (Almannai et al., 2022; Najmabadi et al., 2011; Suleiman et al., 2018).

In this study, we report another new case with a novel variant and clinical features caused by *WDR45B* variant.

## 2 MATERIALS AND METHODS

### 2.1 Ethical compliance

Informed consent was obtained from the proband and her family. This study was approved by the Institutional Review Board of The Second Hospital of Hebei Medical University. Clinical features, brain magnetic resonance imaging (MRI), malformations, and other examination results were collected.

### 2.2 WES and Sanger sequencing

Genomic DNA was extracted from the whole blood sample. The IDT XGen Exome Research Panel was used to capture libraries, and then, the library was sequenced on the NovaSeq 6000 Sequencing platform. Finally, the paired‐end clean reads were compared to the human reference genome (GRCh38/hg38). Variations were annotated through ANNOVAR (Wang et al., 2010), and SNPs with a minor allele frequency of <0.01 in the SNP database were obtained for further analysis. Pathogenic evaluation was performed according to the ACMG (American College of Medical Genetics and Genomics) guidelines (Richards et al., 2015). Sanger sequencing of candidate variants was performed on samples from the proband and her parents to validate the variation identified by WES.

### 2.3 Protein 3D structure

DUTE (http://biosig.unimelb.edu.au/duet/stability_prediction) was utilized to predict the stability of mutant proteins. Molecular modeling analysis was performed to show the variations in protein structure. The human_WDR45B model was downloaded in the AlphaFold dataset (doi:10.1038/s41586‐021‐03819‐2), and Missense3D software was utilized to simulate mutant structure. UCSF Chimera software was used for 3D protein structure visualization and analysis.

## 3 RESULTS

### 3.1 Case presentation

An 8‐year‐old girl was admitted to the hospital due to vomiting, weakness, and poor spirit. She was born at full term, could sit alone at 8 months, crawl at 15 months, walk at 20 months, and only speak overlapping words at two years old; these features suggested global developmental delay. During hospitalization, she could not communicate and walk alone and exhibited spastic quadriplegia, joint contractures, nystagmus, intermittent strabismus, no autonomic movement of limbs, and hypotonia.

**FIGURE 1:**
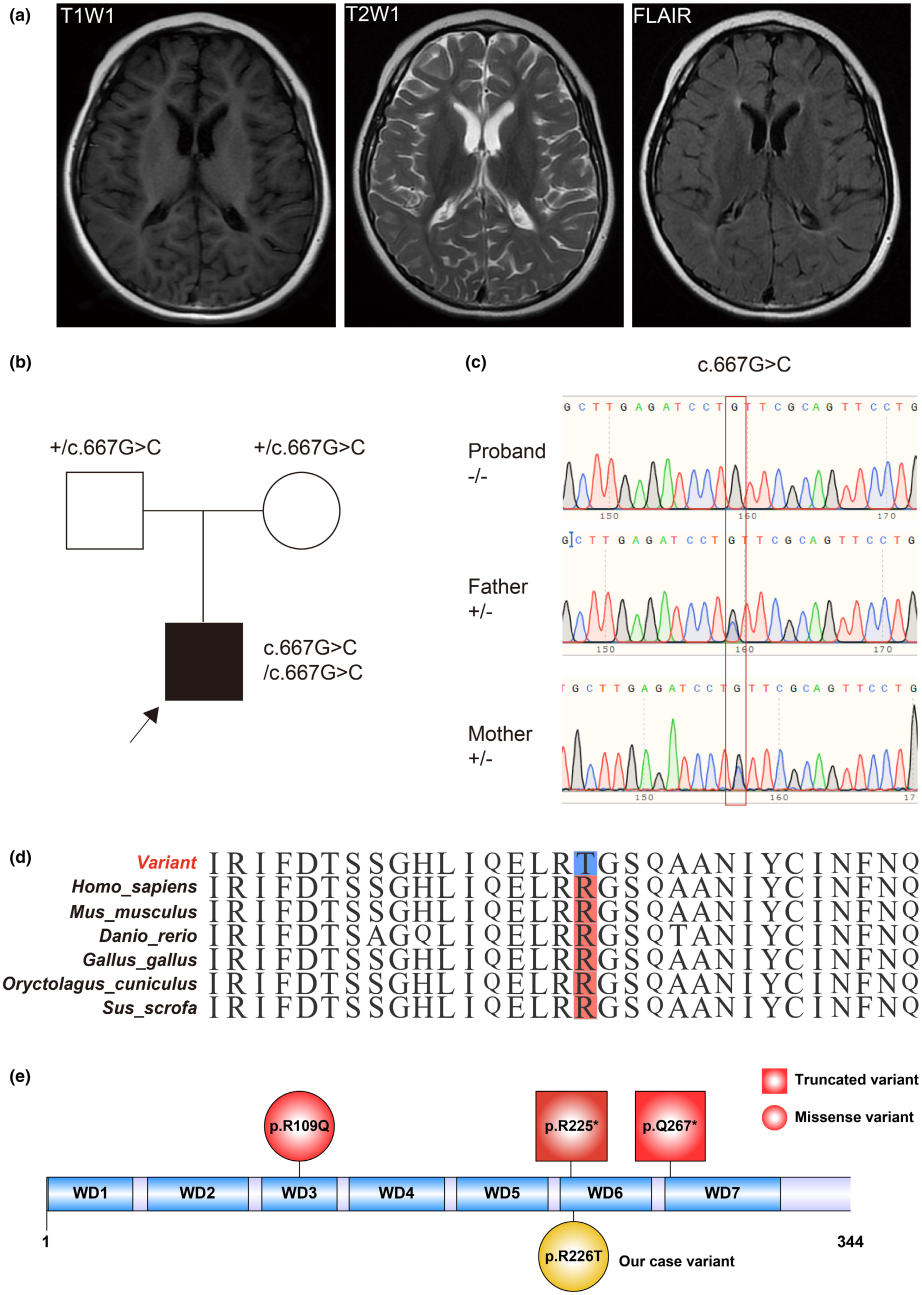
Clinical and genetic features of the proband. (a) Characteristic change of the proband MRI. MRI images showed patchy abnormal signals in the left frontal–parietal lobe and bilateral paraventricles, and high‐density signals in T2 and FLAIR. MRI: magnetic resonance imaging. (b) Pedigree chart and genotype information of the proband. +, wild‐type; Black arrow, proband. (c) Genotype validated by Sanger sequencing. Homozygous variants were inherited from the father and mother. −, variant type; +, wild type. (d) Evolutionary conservation analysis of the 226th amino acid residue. Red highlighted amino acid residue: the wild‐type amino acid residue among multiple species. Blue highlighted amino acid residue: the variant amino acid residue. (e) Variants reported summary. Red square: Reported truncated variants. Red circle: Reported missense variants. Yellow circle: Variants discovered in our case. WD1 ~7: WD repeats 1–7.

Video EEG revealed extensive β‐rhythm at 15–20 Hz during the waking and sleeping stages. Cranial MRI showed multiple abnormal signals in the left frontal–parietal lobe and bilateral paraventricular white matter (Figure 1a).

She was firstly treated with ganciclovir (150 mg twice per day) for antiviral, aceglutamide (200 mg/day) for nerve nutrition, and intravenous immunoglobulin (10 g/day). Then, she was treated with mitochondrial treatment cocktail: Vitamin B1 tablets (30 mg tid); Coenzyme Q10 (100 mg tid); Idebenone (30 mg tid); Vitamin E (100 mg qod); Vitamin B2 (25mg tid); levocarnitine (1 g tid). After that, the vomiting, spirit, and weakness improved, and she was discharged 28 days later.

Two months later, the child still exhibited hypotonia and could not sit or stand alone. The nystagmus was improved. Brain MRI showed a reduced range of multiple abnormal signals in the left frontal–parietal lobe and bilateral paraventricular lobe.

### 3.2 Whole‐exome sequencing, data interpretation, and literature review

To further identify the cause of disease, whole‐exome sequencing (WES) was performed. A homozygous variant in *WDR45B* [NM_019613.4: c.677G>C (p. Arg226Thr)] was found in our patient (Figure 1b). It was a nonsynonymous variant that resulted in an amino acid change from arginine to threonine at the 226th residue of *WDR45B*. The variant was absent in published databases such as gnomAD and Exome Aggregation Consortium (ExAC). The variant was predicted to be damaging to proteins (SIFT: Damaging, Polyphen2 _HDIV: Damaging, Mutation Taster: Disease‐causing) and classified as VUS according to ACMG guidelines. The homozygous variant was confirmed by Sanger sequencing (Figure 1c). Further conservation analysis among multiple species showed that the amino acid was highly conserved (Figure 1d). We reviewed all reported variants of *WDR45B* and found all variants (including our case) located within the repeat WD domains (Figure 1e).

### 3.3 3D protein structure changing

**FIGURE 2:**
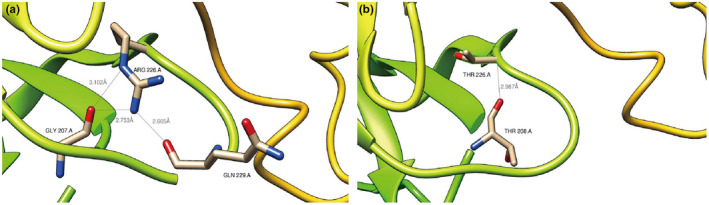
3D protein structure for WDR45B. (a) Wild‐type WDR45B protein structure. (b) R226T protein structure. Amino acids hydrogen bonded to amino acid 226 are highlighted. Hydrogen bond distances were shown.

Protein structural analysis was performed to further explore the effect of variant on protein function. DUET software predicted that the variant R226T destabilizes protein structure with −2.059 Kcal/mol. R226T variant obviously changed the hydrogen bond connection of the 226th amino acid. The hydrogen bond between the 226th amino acid and the original 207, 229th amino acid is broken, and the hydrogen bond is formed with the 208th amino acid (Figure 2). These changes of the structure may tend to be protein unstable.

## 4 DISCUSSION

WD40 repeat was defined as around 40 amino acids which terminated in a tryptophan–aspartic acid (W‐D) dipeptide. WD40 repeat proteins contained four or more WD40 repeat units, which were crucial for forming the circular solenoid domain (WD40 domain) (Smith, 2008). Previously, studies demonstrated WD40 repeat proteins involved in a variety of functions from signal transduction and transcription regulation to cell cycle control, autophagy, and apoptosis (Stirnimann et al., 2010). Notably, some WD40 repeat proteins were reported to be associated with rare disorders, for example, *WDR45* with psychomotor development delay (Saitsu et al., 2013), *WDR35* with cranioectodermal dysplasia (Gilissen et al., 2010), and *WDR56* with short‐rib thoracic dysplasia (Cavalcanti et al., 2011), etc. (Li & Roberts, 2001).

As *WDR45* and *WDR45B* were also reported associated with development delay, for example, J Suleiman et al. described a homozygous pathogenic variant (c.673C>T, p.R225*) in two families. The probands exhibited profound development delay, early‐onset refractory epilepsy, progressive spastic quadriplegia, contractures, and brain malformations (Suleiman et al., 2018); Najmabadi et al. reported a homozygous missense variant (R109Q) from three members of a closely related family with intellectual disability and microcephaly (Najmabadi et al., 2011); Almannai et al. presented 12 individuals from seven unrelated families with microcephaly, global developmental delay, seizures, and spastic quadriplegia (Almannai et al., 2022). These studies demonstrated the role of *WDR45B* in neurodevelopmental diseases.

We reviewed all published cases of *WDR45B* and summarized the clinical features, physical examinations, genetic variants (Table 1), and brain MRI results (Table 2). Compared with the other reported cases, our case showed specific features including multiple abnormal signals around the bilateral paraventricular white matter. No spastic quadriplegia, microcephaly, ventriculomegaly, reduced cerebral white matter volume in the corpus callosum, or thinning of cerebral gray matter was observed in our case.

**TABLE 1:** Clinical features and genetic variants of *WDR45B*‐related cases

Family	No.	Age	Gender	Developmental delay (19/20)	Spastic quadriplegia (15/20)	Epilepsy (17/20)	variants	Variant type	Reference
Family 1	1	16 years	Female	+	+	+	c.799C > T, p.Q267*	Homozygous	PMID: 28,503,735
	2	8 years	Male	+	+	+			
	3	9 months	Female	+	+	+			
Family 2	4	20 years	Male	+	+	+	c.673C > T, p.R225*	Homozygous	
	5	14 years	Female	+	+	+			
Family 3	6	8 years	Female	+	+	−			
Family 4	7	NA	NA	NA	NA	NA		Homozygous	PMID: 21937992
Family 5	8	4 years	Female	+	−	+	c.674G > A, p.R225Q	Homozygous	PMID: 35322404
	9	14 years	Male	+	−	−			
Family 6	10	21 months	Male	+	+	+		Homozygous	
Family 7	11	6 years	Male	+	+	+	c.619‐3A>G	Homozygous	
	12	22 months	Male	+	+	+			
Family 8	13	7 months	Male	+	+	+	c.673C>T, p.R225*	Homozygous	
Family 9	14	8 years	Female	+	+	+	c.427+4A>G	Homozygous	
	15	10 years	Female	+	+	+			
	16	11 years	Male	+	+	+			
Family 10	17	15 years	Female	+	+	+			
	18	3 years	Male	+	+	+			
Family 11	19	3 years	Female	+	NA	+	c.67+1G>T	Homozygous	
Our case	20	8 years	Female	+	−	+	c.677C>T, p.R226T	Homozygous	Our case

**TABLE 2:** Brain development and MRI results of *WDR45B*‐related cases

Family	No.	Age	Gender	Microcephaly (15/20)	Ventriculomegaly (14/20)	Reduced cerebral white matter volume with thin corpus callosum (14/20)	Thinning of cerebral gray matter (15/20)	Reference
Family 1	1	16 years	Female	−	+	+	+	PMID:28503735
	2	8 years	Male	−	+	+	+	
	3	9 months	female	−	+	+	+	
Family 2	4	20 years	Male	+	NA	NA	NA	
	5	14 years	Female	+	+	+	+	
Family 3	6	8 years	Female	+	+	+	+	
Family 4	7	NA	NA	NA	NA	NA	NA	PMID: 21937992
Family 5	8	4 years	Female	+	+	+	+	PMID: 35322404
	9	14 years	Male	+	−	−	+	
Family 6	10	21 months	Male	+	NA	NA	NA	
Family 7	11	6 years	Male	+	+	+	+	
	12	22 months	Male	+	+	+	+	
Family 8	13	7 months	Male	+	+	+	+	
Family 9	14	8 years	Female	+	+	+	+	
	15	10 years	Female	+	+	+	+	
	16	11 years	Male	+	+	+	+	
Family 10	17	15 years	Female	+	NA	NA	NA	
	18	3 years	Male	+	+	+	+	
Family 11	19	3 years	Female	+	+	+	+	
Our case	20	8 years	Female	−	−	−	−	Our case

## AUTHOR CONTRIBUTIONS

Jinhong Zhang: Conceptualization, Methodology, Data mining; Yan Lu, Xiaoyu Tian: Clinical data collection; Xinyi Men, Yange Zhang, Huifang Yan: Writing‐ Original draft preparation; Zuozhen Yang, Fan Yang: Software, Data interpretation; Xiuxia Wang: Supervision, Writing‐ Reviewing and Editing.

## Funding information

This work was supported by the Medical Science Research Project Plan of Hebei Province (No. 2019078).

## CONFLICT OF INTEREST

All authors declare no conflicts of interest.

## ETHICS STATEMENT

All procedures performed in this study involving human participants were in accordance with the Declaration of Helsinki (as revised in 2013). This study was approved by the institutional review board of The Second Hospital of Hebei Medical University. Informed consent was obtained from the proband and her family.
